# The Beneficial Outcome of Subsequent Treatment with Anakinra during the Chronic Phase of Febrile Infection-Related Epilepsy Syndrome (FIRES): A Case Report

**DOI:** 10.3390/neurolint15040097

**Published:** 2023-12-11

**Authors:** Tina Luize Cupane, Jurgis Strautmanis, Signe Setlere, Mikus Diriks, Madara Auzenbaha

**Affiliations:** 1 Faculty of Medicine, University of Latvia, LV-1004 Riga, Latvia; 2 European Reference Network EURO-NMD, 75013 Paris, France; signe.setlere@bkus.lv (S.S.); mikus.diriks@bkus.lv (M.D.); 3 Epilepsy and Sleep Medicine Centre, Children’s Clinical University Hospital, LV-1004 Riga, Latvia; jurgis.strautmanis@bkus.lv; 4 European Reference Network Epi-CARE, 69677 Bron, France; 5 Neurology and Neurosurgery Department, Children’s Clinical University Hospital, LV-1004 Riga, Latvia; 6 Medical Genetics and Prenatal Diagnostic Clinic, Children’s Clinical University Hospital, LV-1004 Riga, Latvia; madara.auzenbaha@bkus.lv

**Keywords:** FIRES, anakinra, IL-1 receptor antagonist, immune-modulating therapy

## Abstract

This case report presents the clinical course of an eight-year-old boy diagnosed with febrile infection-related epilepsy syndrome (FIRES) at the age of four. Following a febrile infection, the patient experienced his initial episode of serial generalized clonic seizures. The severity of his condition led to 11 hospital admissions, totaling 157 days of hospitalization. Anakinra was initially administered during the acute phase in 2019 but was discontinued after 29 days. In 2022, the patient experienced a chronic-phase exacerbation and underwent a second course of anakinra treatment, which demonstrated a positive effect on seizure activity. With a year of anakinra therapy, the patient exhibited significant improvement in both seizure frequency and severity. This report adds to the existing evidence supporting the potential use of anakinra in the treatment of FIRES, highlighting its effectiveness during the chronic phase and suggesting the potential benefits of subsequent administration.

## 1. Introduction

FIRES (febrile infection-related epilepsy syndrome) is a disorder in which refractory status epilepticus occurs within the first 24 h to 2 weeks after a febrile infection, with or without fever at the onset of status epilepticus (SE). Previously healthy children (aged 5 to 13) experience recurrent seizures and SE during the acute phase, leading to persistent seizures and cognitive impairment [1]. The pathogenesis remains unknown and could be considered as an immune system dysregulation that manifests in healthy individuals [2,3]. FIRES is a subcategory of NORSE (new-onset refractory status epilepticus), a condition characterized by sudden refractory SE without an identifiable structural, toxic, or metabolic cause [4]. The global incidence is approximately 1 in 1,000,000. Over 220 FIRES cases have been published from 1961 to 2022 [5].

FIRES presents in two phases. The acute phase is characterized by seizure activity of up to 12 weeks [3]. Seizures intensify in a crescendo-like pattern. Over the course of one week, they tend to increase in frequency and duration, eventually developing into refractory status epilepticus, with little to no response to traditional antiseizure medications [6]. The electroencephalogram (EEG) of children can exhibit recurring characteristics that include extreme delta brush, escalating seizure frequency, and focal seizure activity with shifting low-amplitude fast activity between brain hemispheres before culminating contralaterally [7]. The seizure activity tends to decrease, leading to gradual recovery. Patients who survive are mostly left with considerable cognitive deficits and chronic intractable epilepsy [8,9]. The cognitive outcomes vary: One-third have normal/borderline results, one-third have mild-to-moderate disability, and the rest experience severe disability/vegetative state [10]. The prognosis depends on the acute-phase duration, with up to 30% fatality [3]. The chronic phase involves clusters of refractory seizures every 2–4 weeks, manifesting as a neurological sequela that presents with both cognitive and language impairment, as well as motor dysfunction and drug-resistant seizures [2].

There are only a limited number of approaches for treating FIRES, including antiseizure medications, anakinra (interleukin-1 receptor antagonist), cannabidiol, tocilizumab (anti-IL-6 therapeutic agent), canakinumab (anti-IL-1β), rituximab (anti-CD20 monoclonal antibody), and the ketogenic diet [3,4,11,12,13]. It has been hypothesized that administering anakinra during the acute phase of the disorder could decrease the burden of seizures [14,15]. The early initiation of anakinra has been linked to reduced mechanical ventilation days and a shorter length of stay in the intensive care unit and hospital [14]. High-dose steroids, intravenous immunoglobulin (IVIG), have been reported to be effective in certain cases [3,16]. Therapeutic hypothermia proves to have anti-inflammatory and neuroprotective properties, which in some cases might help to reduce the seizure burden [3]. The chronic phase presents significant treatment challenges, and on rare occasions, patients can become seizure-free. The use of cannabidiol, anakinra, rituximab, or in some cases, epilepsy surgery has been recommended. However, the efficacy of these approaches remains a subject of ongoing discussion [5].

We present a case of an 8-year-old with FIRES and intractable epilepsy, showing a favorable response to anakinra during the chronic phase. Over the course of one year, there was a progressive reduction in seizure severity and frequency. This case adds valuable evidence to the current research on the efficacy of anakinra in the chronic phase of FIRES.

## 2. Methods

The parents signed a consent form that followed the Helsinki Declaration principles beforehand.

We analyzed the demographic and clinical data at Children’s Clinical University Hospital, with ethical approval from the University of Latvia’s Research Ethics Committee (Nr. 19-25/85), on 22 February 2023.

## 3. Case Description

We present the case of a previously healthy boy who, at the age of four, was diagnosed with FIRES. It began in July 2019 when his initial symptoms manifested following a challenging-to-treat high fever (39 °C). On Day 4, he experienced generalized tonic–clonic seizures that did not respond to diazepam, eventually necessitating a transfer to a tertiary health center.

**Figure 1:**
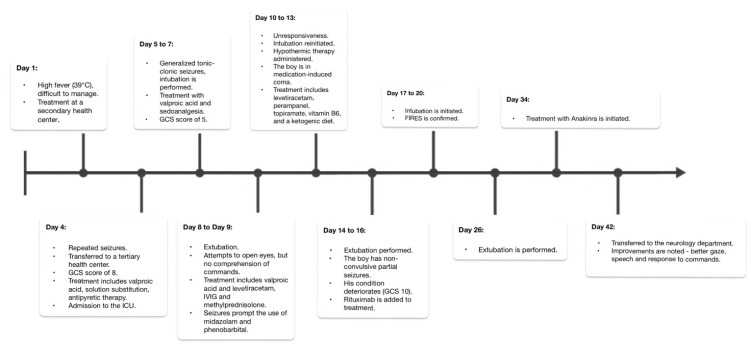
The timeline of FIRES in a 4-year-old boy (Day 1 to ICU discharge).

Upon admission, his Glasgow Coma Scale (GCS) score was 8, and he exhibited eyeball floating and increased muscle tone. Treatment was initiated with a valproic acid infusion, fluid replacement therapy, and antipyretics to manage his condition. He was transferred to the intensive care unit (ICU), where comprehensive analyses were conducted to rule out viral and bacterial infections. In the following days, he experienced generalized tonic–clonic seizures; was intubated; and received treatment with valproic acid, midazolam, and fentanyl. A lumbar puncture was performed, confirming aseptic meningitis (18 cells/microliter). The brain MRI revealed acquired periventricular white matter changes in the frontal lobes. His condition worsened, with a GCS score of 5 and generalized tonic–clonic seizures, leading to treatment with methylprednisolone (500 mg) and intravenous immunoglobulin (IVIG) therapy (0.4 g/kg daily for 5 days) due to the suspicion of autoimmune encephalitis. Extubation was attempted, and thiopental was decreased. Although he attempted to open his eyes, he did not comprehend commands. The generalized tonic–clonic seizures prompted the use of midazolam and phenobarbital, and despite these interventions, he became unresponsive. The seizures persisted, characterized by breath holding and eye opening for up to 1 min and 30 s. Intubation was reinitiated, hypothermic therapy was administered, and a medication-induced coma was induced. Extensive tests, including those for autoimmune and oncological markers, as well as genetic analysis through trio whole-exome sequencing (WES), were conducted and later yielded negative results. EEG recordings revealed background slowing, prominent slow wave activity in the right temporal region, and multifocal epileptiform activity predominantly in both temporal regions. Brain MR spectroscopy indicated prior hypoxia. Extubation followed, leading to frequent nonconvulsive partial seizures. On Day 14, his condition deteriorated (GCS 10), with intensified seizures. On Day 16, rituximab (260 mg) was added to the treatment. Further intubation and treatment followed, supplemented by baclofen and clonidine. FIRES was confirmed on Day 20. Extubation was performed on Day 26, and anakinra (50 mg/day) was started on the 34th day as the acute phase treatment for FIRES. After a 42-day ICU stay, he was transferred to the neurology department (Figure 1). During his ICU stay, he received thiopental (6 days), fentanyl (10 days), midazolam (26 days), baclofen (17 days), clonidine (16 days), clonazepam (17 days), morphine (15 days), and quetiapine prior to transfer. After discharge from the ICU, improvements were observed, including better gaze, speech, and response to commands. Hyperkinesis appeared as excessive and involuntary movements. Within three days, focal seizures began, spreading to one side of the body, causing unconsciousness. The pattern varied, mostly at night, lasting 2–3 days, followed by seizure-free days. The seizures shifted to involve eye fixation and limb twitching, happening 2–6 times/day. On the 48th day, the MRI revealed a widened brain sulci, enlarged lateral ventricles, and periventricular demyelination—typical of acute FIRES. EEG showed multifocal epileptiform activity.

In September, after 29 days, treatment with anakinra was stopped due to uncertainty about the efficacy of this treatment. In October, focal seizures occurred up to twelve times per day, affecting one side of his body and occasionally resulting in loss of consciousness. His activity level increased, and he was able to take a few steps. The Atkins diet replaced the ketogenic diet. After 118 days, his condition stabilized, reducing the seizure frequency and intensity (up to three times/day). Atonic seizures caused falls. Cannabidiol was added, following the mother’s suggestion. After 119 days, he was discharged.

Throughout 2020, he experienced up to seven seizures per day. He was walking, running, jumping, and climbing stairs. Verbal communication remained challenging. Follow-up MRI scans showed no notable changes; EEG results indicated multifocal epileptic activity. He exhibited no significant changes in his condition until August 2021, when he was admitted to the hospital due to status epilepticus. The seizure frequency exceeded 48 times a day and lasted up to 1 min. The symptoms included eye opening, dilated pupils, sequential limb extension, and flexion of the right arm. Following phenobarbital and valproic acid administration, the seizures subsided, and he was discharged. In March 2022, his condition became worse after contracting COVID-19, as well as an unspecified bacterial infection. The seizures began with facial twitching; then, they were generalized, lasting 1 min. The frequency increased with a new seizure semiology (temporary stiffness and fixed gaze after generalized seizures). Verbal communication was impossible. He was admitted to the ICU and diagnosed with polysinusitis and sphenoiditis. After sphenoidectomy and antibacterial treatment, he faced seizure clusters with eye, head, and limb involvement, lasting up to 1 min, persisting for 2–3 h. Alongside medications (sultiam, phenobarbital, cannabidiol, brivaracetam, and melatonin), due to his severe condition that did not respond to treatment, anakinra was reintroduced for the second time. His condition was stable. He was conscious, spontaneously opened his eyes, and did not respond to commands or exhibit verbal expressions. The seizure intensity and frequency decreased (horizontal eye movements and head turning) and lasted up to 1 min, and the patient was discharged from the hospital with the ability to sit up, briefly stand with a support, and move his eyes and limbs.

**Figure 2:**
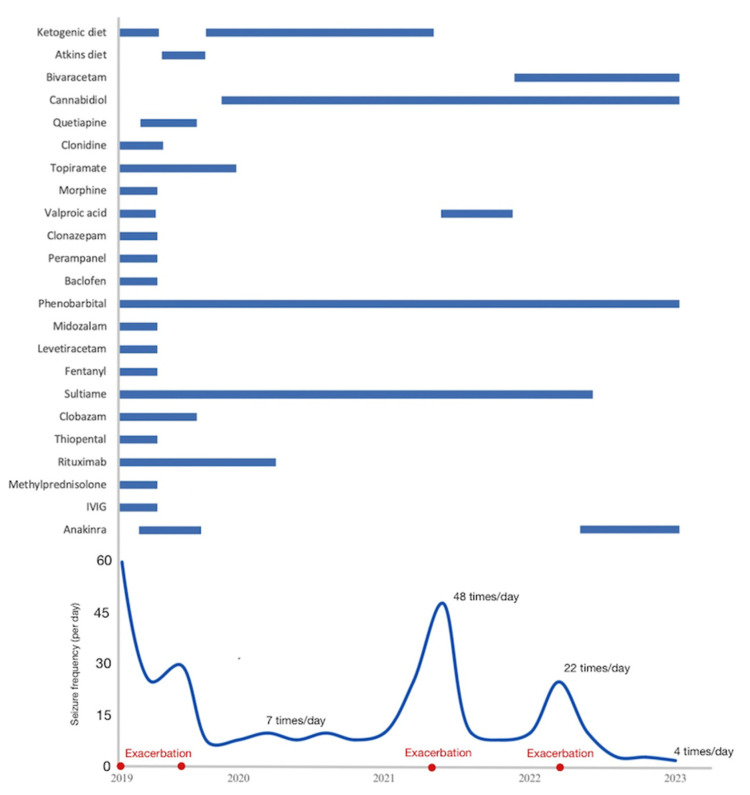
Treatment progression, exacerbation events, and seizure frequency (2019–2023).

In the chronic phase of FIRES, anakinra was once again introduced as part of the therapeutic regimen. By June 2022, a significant decrease in seizure frequency was observed, with up to 10 seizures per month, marked by episodes of eye deviation, fixation, and facial spasms, each lasting approximately 1 min. He demonstrated independent movements and a partial understanding of commands; verbal communication remained absent, consistent with his condition before March. He regained the ability to go for walks. In addition to anakinra, the patient had been on phenobarbital and brivaracetam for over a year. This comprehensive treatment approach contributed to his gradual improvement (Figure 2).

## 4. Discussion

We present the case of an 8-year-old boy with FIRES who underwent anakinra treatment during the chronic phase. He experienced four exacerbations, with the most severe occurring at the disease’s onset and three years later, following a contraction of COVID-19. The patient endured a total of 11 hospital admissions, amounting to 157 days in the hospital. While extensive research delves into the acute phase of FIRES, our understanding of the chronic phase is limited, posing challenges in finding effective treatments. A recent study with seven FIRES patients provided insights into the chronic phase, revealing that in nearly all cases, it occurred without an intervening silent period following the acute phase. Seizures during the chronic phase were resistant to most anticonvulsant medications [17], mirroring our patient’s experience. In other cases, treatment during the chronic phase has involved using cannabidiol. This approach led to a significant decrease in seizure frequency and improvements in motor skills, cognitive function, and verbal abilities [11]. However, our patient had already undergone this treatment before the last exacerbation, which made it a less suitable option. Highlighting the variety of treatment choices and their results can help us grasp the strategies required to handle FIRES. In a case study involving a 4-year-old patient experiencing unusual symptoms during the chronic phase of FIRES, including dystonia, multiple treatment approaches were attempted. They included various antiseizure medications, continuous phenobarbital infusion, glucocorticoids, intravenous immunoglobulin, plasmapheresis, anakinra treatment, and cannabidiol. None of these treatments resulted in positive outcomes, and the patient developed severe encephalopathy [18]. By contrast, our patient faced significant cognitive impairment after the disease onset, despite implementing the appropriate treatment later on.

Determining whether these impairments were due to polytherapy or prolonged status epilepticus that caused permanent effects on the brain is a difficult challenge. However, cognitive disorders in children with epilepsy are common and can range from mild to profound intellectual disability [19].

Attaining remission from status epilepticus proved challenging from the beginning. During the acute phase, we administered antiseizure medications, including valproic acid and midazolam. Subsequently, we induced a barbiturate coma to achieve burst suppression and control the seizures. However, this led to complications, such as respiratory distress. In similar cases, anesthetic agents like midazolam infusion, pentobarbital, or a combination thereof are often required to control seizures before initiating anakinra treatment [6,20]. Many patients, ours included, have faced failures with various antiseizure medications, as well as both first- and second-line immunotherapies, before turning to anakinra. Although anakinra often provides benefits from the outset in many patients, our case was different [1,2,3]. The initial omission of anakinra did not lead to the deterioration of the patient’s condition, causing us to question its efficacy. Some reports indicate that anakinra treatment was ineffective [18], prompting a switch to tocilizumab [21]. Treatment approaches during the chronic phase primarily aim at symptom management. This often involves multiple medications, with standard first-line treatments including a ketogenic diet, clobazam, phenobarbital, and possibly cannabidiol [18,21]. In our case, these treatments were ineffective during the most recent exacerbation in 2022. To make an informed decision, we reintroduced anakinra in the chronic phase—an aspect less explored in research, especially when the treatment was initially halted and then restarted. This decision significantly improved the boy’s condition, underscoring the potential benefits of revisiting previously attempted treatments. The patient experienced a marked reduction in seizure frequency, regained independent movement, began to partially understand commands, and relearned walking. It is crucial to consider that other medications might have influenced his response to anakinra. However, pinpointing the exact impact is challenging and remains speculative. The patient was also treated with brivaracetam, which is known for its seizure-reducing properties [6,11]. Supporting our findings, a study involving five patients in the chronic phase was published. It detailed their experiences with either anakinra or tocilizumab. Six months later, three of these patients experienced a 50% drop in seizure frequency, while the others had milder seizures, relied less on rescue medications, and exhibited notable improvements in behavior and communication [22].

The first time, we administered anakinra treatment for a duration of 29 days. However, it was only upon reintroducing it three years later that the patient showed marked improvement. This situation raises questions: Would a longer anakinra treatment period have been more effective? What is the ideal treatment duration? Determining the optimal timing for anakinra treatment remains a challenge. An early start appears promising for enhanced outcomes and potential seizure reduction [18]. Yet, the exact duration remains ambiguous. A case series that explored the efficacy of anakinra and tocilizumab during the chronic phase of FIRES shed light on the usage of anti-IL1 and anti-IL-6 therapies. The series reported that the median treatment duration for anakinra was 9 months, starting with a daily dosage of 100 mg. The timing for initiating the treatment varied, with the median being 7 years after the acute phase of FIRES. Noticeable improvements in seizure outcomes were observed within 2–3 weeks of starting the treatment. Interestingly, those who responded most significantly to anakinra had waited a considerable time after the acute phase before beginning treatment—up to 11 years in some cases [22]. This suggests that even a delayed treatment initiation can yield positive outcomes. In contrast, Dilena et al. described a case where anakinra was administered during the chronic phase for 1.5 years [23]. Similarly, Yang et al. presented a case of a previously healthy six-year-old girl who underwent a year of anakinra treatment. This treatment led to positive results, characterized by shorter and less frequent seizures [24]. In our patient’s scenario, a year of anakinra treatment, accompanied by antiepileptic drugs, dramatically enhanced seizure control following four years of hospitalization. Generally, patients tolerate anakinra well, and it is effective in treating FIRES and refractory epilepsy [21].

Common side effects include local reactions at the injection site, leukopenia, and increased transaminases. Serious infections are rare [25], and our patient experienced no side effects, reinforcing anakinra as a favorable treatment option. However, whether it is suitable for lifelong treatment remains uncertain. More research is essential to establish the best duration for anakinra therapy.

## 5. Conclusions

This patient experienced up to seven daily seizures and severe exacerbations four times from 2019 to 2022. Upon starting anakinra in the chronic phase, seizures were reduced to four per day without any subsequent exacerbations. This suggests that anakinra impacts seizure/status epilepticus frequency in the chronic phase. We recommend considering a second round of anakinra for those in the chronic phase of FIRES. Further research is essential to determine the safety and effectiveness of this approach and to inform clinical practice guidelines.

## Data Availability

The data generated during and/or analyzed during the current study are available from the corresponding author upon reasonable request. Access to the data is subject to any ethical or legal restrictions imposed by the relevant research institutions or governing bodies. To obtain the data, interested parties can contact the corresponding author, Tina Luize Cupane, via e-mail at tlcupane@gmail.com. The corresponding author will provide the data promptly and ensure compliance with any applicable data protection and privacy regulations.
